# Facile Route for Bio-Phenol Siloxane Synthesis via Heterogeneous Catalytic Method and its Autonomic Antibacterial Property

**DOI:** 10.3390/polym10101151

**Published:** 2018-10-16

**Authors:** Xiaoyan Pang, Xin Ge, Jianye Ji, Weijie Liang, Xunjun Chen, Jianfang Ge

**Affiliations:** 1 College of Chemistry and Chemical Engineering, Zhongkai University of Agriculture and Engineering, Guangzhou 510225, China; shelly_pxy@163.com (X.P.); gexin@zhku.edu.cn (X.G.); jjyjasonky@163.com (J.J.); cxj.qiao@163.com (X.C.).; 2 School of Materials Science and Engineering, Northwestern Polytechnical University, Xi'an 710072, China; leungvijer@163.com

**Keywords:** bio-phenol siloxane, synthesis, supported platinum catalyst, autonomic antibacterial

## Abstract

Eugenol, used as bio-phenol, was designed to replace the hydrogen atom of hydrogenterminated siloxane by hydrosilylation reaction under the presence of alumina-supported platinum catalyst (Pt-Al_2_O_3_), silica-supported platinum catalyst (Pt-SiO_2_) and carbon nanotube-supported platinum catalyst (Pt-CNT), respectively. The catalytic activities of these three platinum catalysts were measured by nuclear magnetic resonance hydrogen spectrometer (^1^H NMR). The properties of bio-phenol siloxane were characterized by Fourier transform infrared spectrometer (FT–IR), UV-visible spectrophotometer (UV) and thermogravimeter (TGA), and its antibacterial property against *Escherichia coli* was also studied. The results showed that the catalytic activity of the catalyst Pt-CNT was preferable. When the catalyst concentration was 100 ppm, the reaction temperature was 80 °C and reaction time was 6 h, the reactant conversion rate reached 97%. After modification with bio-phenol, the thermal stability of the obtained bio-phenol siloxane was improved. For bio-phenol siloxane, when the ratio of weight loss reached 98%, the pyrolysis temperature was raised to 663 °C which was 60 °C higher than hydrogenterminated siloxane. Meanwhile, its autonomic antibacterial property against *Escherichia coli* was improved significantly.

## 1. Introduction

A bio-phenol is a phenol that derives from natural products, for example, vegetablse, tea-leaves, and flowers. Eugenol (4-allyl-2-methoxyphenol) is one kind of bio-phenol, which is a primary constituent of plant essential oil such as clove oil, laurel oil and camphor oil and characterized by the presence of functional groups such as hydroxy group, methoxy group and allyl group. Because of this unique structure, eugenol has biological and chemical characteristics, for example, antibacterial activity, antioxidant activity thermal stability and chemical reactivity [1,2,3,4]. Eugenol is particularly widely used in the food, cosmetic, and pharmaceutical industries [5,6]. Meanwhile, eugenol was used in compound modification [7,8]. Dai [9] used eugenol in the synthesis of eugenol-based organic coatings and the results indicated that the coating showed outstanding thermal stability and excellent adhesion. Miao [10] used eugenol in the synthesis of bio-based epoxy resin and the results showed the product had outstanding comprehensive performances and high renewable carbon content. Mangeon [11] used eugenol in the synthesis of semi-interpenetrating polymer networks and used it as plasticizer of poly-(3-hydroxyalkanoate), and the results showed the compound exhibited excellent deformability. Silva [12] studied eugenol derivatives which had excellent antibacterial and antioxidant activities.

Eugenol could replace the hydrogen atom of hydrogenterminated siloxane by hydrosilylation reaction between allyl group and silicon hydrogen group under the presence of platinum catalyst. The platinum catalysts were commonly homogeneous catalysts such as Speier’s catalyst and Karsted’s catalyst [13,14,15]. The homogeneous catalyst was corrosive to reactors, difficult to remove from the system and had poor selection of reaction, and thus limited the application in specific fields [16,17]. Recently, the supported platinum catalysts are attracting researchers’ interest because of excellent catalytic selectivity and their simplicity of removal [18,19,20,21,22]. It is notable that supported catalysts have different catalytic activities in the reaction because of the different properties of supported material.

In this paper, eugenol, used as a bio-phenol, was designed to replace the hydrogen atom of hydrogenterminated siloxane by hydrosilylation reaction under the presence of alumina-supported platinum catalyst (Pt-Al_2_O_3_), silica-supported platinum catalyst (Pt-SiO_2_) and carbon nanotube-supported platinum catalyst (Pt-CNT). The catalytic activities of these three catalysts were measured by nuclear magnetic resonance hydrogen (^1^H NMR) spectrometer, thereby determining the preferable process conditions. The product, bio-phenol siloxane, was characterized by Fourier transform infrared spectrometer (FT–IR), ultravioler–visible (UV) spectrophotometer and thermogravimeter, and its antibacterial activity against *Escherichia coli* was studied.

## 2. Materials and Methods 

### 2.1. Materials

Eugenol of 98% purity was purchased from Guangdong Tongcai New Material Corporation (Guangzhou, China). Octamethylcyclotetrasiloxane (D_4_) and 1,1,3,3-tetramethyldisiloxane (HMMH) were obtained from Shenzhen Ji-Peng Silicon Fluoride Materials Corporation (Shenzhen, China). Macroporous cationic resin was gained from Jiangyin Nanda Synthesis Chemical Corporation (Jiangyin, China). Catalyst Pt-Al_2_O_3_ with 3% Pt and catalyst Pt-SiO_2_ with 3% Pt were obtained from Hubei Xinrunde Chemical Corporation (Wuhan, China). Chloroplatinic acid was purchased from Shanghai SSS Reagent Corporation (Shanghai, China). CNT was obtained from Qinhuangdao ENO High-Tech Material Development Corporation (Qinhuangdao, China). Agar nutrient solution and *Escherichia coli* solution were prepared from biochemical laboratory in Zhongkai University of Agriculture and Engineering. Ethanol (AR) was supplied by Tianjin Kemiou Chemical Corporation (Tianjin, China).

### 2.2. Synthesis of Bio-Phenol Siloxane

CNT (2.0 g) and ethanol (35 mL) were added into a 100 mL three-necked flask equipped with a magnetic stirrer, a nitrogen inlet and a condenser. After venting nitrogen for 30 min, 15 mL chloroplatinic acid-ethanol solution was added. Then the system was heated to 75 °C for 10 h. After washed and dried, the catalyst Pt-CNT was prepared successfully and the concentration of Pt was 3%.

D_4_ (122.1 g, 0.4125 mol), HMMH (4.02 g, 0.03 mol) and macroporous cationic resin (3.78 g, 3%) were added into a 250 ml three-necked flask equipped with a magnetic stirrer, a thermometer and a reflux condenser. The system was heated to 80 °C for 4 h then purified at 150 °C for 4 h. The hydrogenterminated siloxane was synthesized.

**Figure 1:**
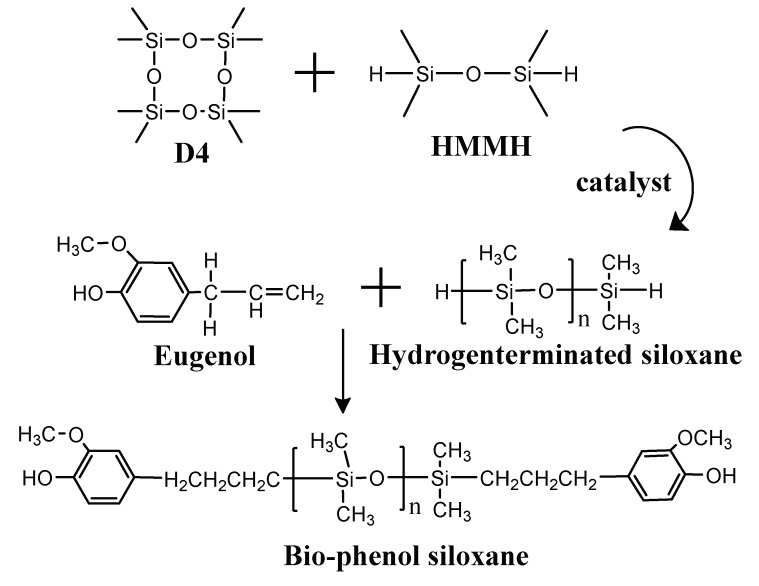
Scheme of synthesis of bio-phenol terminated organosiloxane.

Hydrogenterminated siloxane (80 g, 0.02 mol) and eugenol (6.56 g, 0.04 mol) were placed in a 150-mL three-necked flask equipped with a magnetic stirrer, a nitrogen inlet, and a condenser. After venting nitrogen for 30 min, the appropriate amount of catalyst was added into the three-necked flask, and then the reaction system was heated to set temperature under nitrogen atmosphere for 6 h. Then, after centrifugation for 1 h, viscous oil was obtained. The viscous oil is bio-phenol siloxane. Figure 1 showed the scheme of synthesis of bio-phenol siloxane.

### 2.3. Nuclear Magnetic Resonance Hydrogen (^1^H NMR) Spectrometer Analysis

The ^1^H NMR spectrum were recorded on a Bruker Avance IIIHD 500 spectrometer (Bruker Corporation, Karlsruhe, Germany) using CDCl_3_ as a solvent. The chemical shifts relative to tetramethylsilane, which is used as an internal reference. The yield was calculated by the integration intensity of absorption peak of C=CH_2_ or Si–H.

### 2.4. Fourier Transform Infrared (FT–IR) Analysis

FT–IR spectrum of hydrogenterminated siloxane and bio-phenol siloxane were conducted on a Perkin Elmer FT–IR spectrum 100 (Perkin-Elmer Corporation, Fremont, CA, USA) at room temperature. Before scanning, the samples were dried in the drying cabinet at 80 °C for 4 h to remove moisture. The samples were scanned within the range from 400 to 4000 cm^−1^.

### 2.5. Ultraviolet–Visible (UV) Analysis

UV-vis spectra were recorded on a UV-1800 UV-vis spectrophotometer (Perkin-Elmer Corporation, Fremont, CA, USA) within the range from 200 to 400 nm. The samples were dissolved in the ethanol.

### 2.6. Thermal Analysis

Thermogravimetric analyses were carried out using a Mettler Toledo TG/DTA thermal analyzer (Mettler-Toledo AG Corporation, Columbus, OH, USA) to measure the temperature of siloxane from the beginning of weight loss to 100% weight loss. The experiments were performed in the range of 40–900 °C at a heating rate of 10 °C/min under nitrogen atmosphere.

### 2.7. Antimicrobial Activity

Antimicrobial activity was measured according to a spread plate method using *Escherichia coli* [23]. The amounts of *Escherichia coli* used for the assay were optimized to better visualize the antimicrobial effect caused by eugenol, hydrogenterminated siloxane and bio-phenol siloxane. The agar plates with *Escherichia coli* were placed into the biochemical incubator at 35 °C for 22 h. Optical images of incubated plates were taken with a mobile phone. The amounts of *Escherichia coli* bacterial colonies were observed by manual counting.

## 3. Results and Discussions

### 3.1. Analysis of Catalytic Activity

#### 3.1.1. Catalytic Activity of Pt-Al_2_O_3_

**Figure 2:**
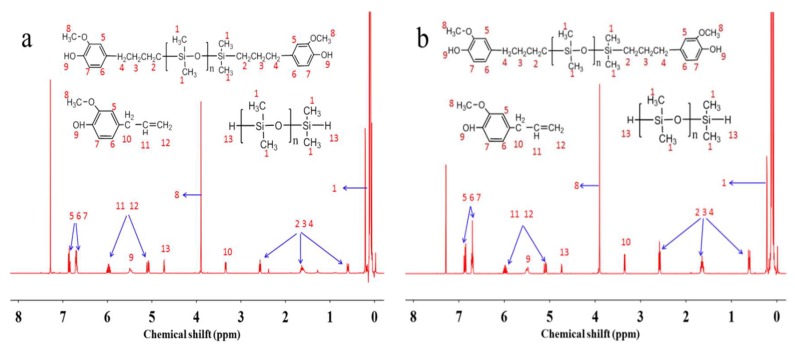
Nuclear magnetic resonance hydrogen (^1^H NMR) spectrum of sample A-2 (**a**) and A-10 (**b**).

Table 1 shows the effect of reaction temperature on activity of the catalyst Pt-Al_2_O_3_ when the amount of catalyst was 100 ppm. The reaction time was set to 6 h in all experiments. As shown in the Table 1, when the reaction temperature was 80 °C, the product was stratified and this explains the existence of unreacted eugenol which was yellowish. By raising the reaction temperature, the degree of reaction improved, and the yellowing degree of product also enhanced, which was detrimental. When the temperature was increased to 160 °C, the product turned into yellow turbid liquid. As the result shows, the preferable temperature for catalyst Pt-Al_2_O_3_ was 100 °C and the sample A-2 was preferable. Figure 2a shows the ^1^H NMR spectrum of sample A-2. As the Figure 2a shown, the signals at 5.9, 5.1, and 4.7 ppm were corresponding to C=CH–, –C=CH_2_ and Si–H group. The ratio of the integration intensity of these three signals is 1:2:1, which matched well with the value calculated according to the structure. This indicated that the reaction system still had unreacted C=CH–, –C=CH_2_ and Si–H group and the yield was 36% by calculating the integration intensity of signal of the Si–H group. Table 2 shows the effect of catalyst concentration on the activity of catalyst Pt-Al_2_O_3_ when the reaction temperature was 100 °C. As Table 2 shown, when the catalyst concentration was increased to 500 ppm, the product was still turbid liquid (Sample A-10). Figure 2b shows the ^1^H NMR spectrum of sample A-10 and we can see the signals of unreacted C=CH–, –C=CH_2_ and Si–H group. The yield was 76%. As known, the turbidity of product demonstrated the reaction degree of system. When the yield was as high as possible, the product was transparent. It was revealed that the catalyst Pt-Al_2_O_3_ had a low catalytic activity in this hydrosilylation reaction between eugenol and hydrogenterminated siloxane.

#### 3.1.2. Catalytic Activity of Pt-SiO_2_

**Figure 3:**
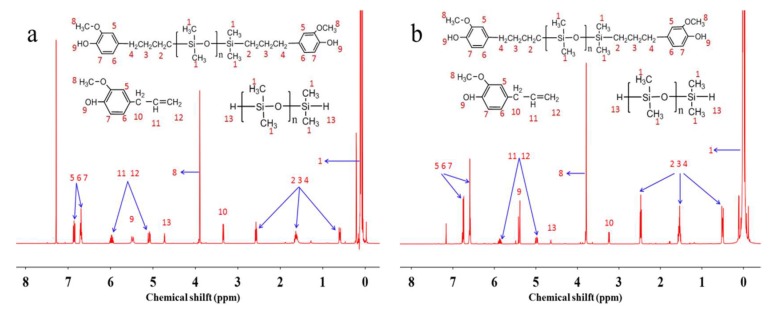
^1^H NMR spectrum of sample B-3 (**a**) and B-7 (**b**).

Table 3 shows the effect of reaction temperature on activity of catalyst Pt-SiO_2_ when the amount of catalyst was 100 ppm. We knew that as the temperature exceeded 100 °C, the product would be turn into yellow, so the temperature was setting at range of 60–100 °C. As Table 3 shown, when temperature was 60 °C, the solution was stratified. When the temperature increased to 100 °C, the solution had turned into yellowish (Sample B-3). Figure 3a is the ^1^H NMR spectrum of sample B-3 and its yield was 66% by calculating the integration intensity of signal of Si–H group. Table 4 shows the effect of catalyst concentration on activity of catalyst Pt-SiO_2_ when the reaction temperature was 80 °C. As Table 2 shown, when the catalyst concentration was increased to 200 ppm, the product was colorless and slight transparent liquid (Sample B-7). Figure 3b is the ^1^H NMR spectrum of sample B-7 and its yield was 80% by calculating the integration intensity of signal of Si–H group. It was revealed that catalyst Pt-SiO_2_ had a higher catalytic activity than catalyst Pt-Al_2_O_3_ in this hydrosilylation reaction. But catalytic activity of catalyst Pt-SiO_2_ was not high enough to meet production requirements.

#### 3.1.3. Catalytic Activity of Pt-CNT

**Figure 4:**
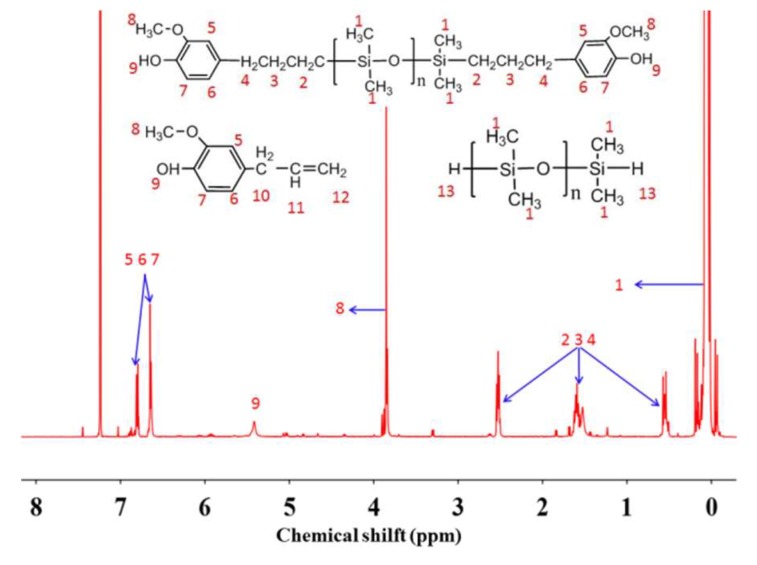
^1^H NMR spectrum of sample C-2.

Table 5 shows the effect of reaction temperature on activity of catalyst Pt-CNT when the amount of catalyst was 100 ppm and the temperature was set at a range of 60–100 °C. As Table 5 shows, when temperature was 60 °C, the solution was colorless and a slightly transparent liquid. When the temperature increased to 80 °C, the solution was a colorless transparent liquid (Sample C-2). Figure 4 is the ^1^H NMR spectrum of sample C-2. As Figure 4 shown, the signals at 5.9, 5.1, and 4.7 ppm had nearly disappeared which were corresponding to C=CH–, –C=CH_2_ and Si–H group. The yield was 97% by calculating the integration intensity of signal of the Si–H group. Table 6 shows the effect of catalyst concentration on activity of catalyst Pt-CNT when the reaction temperature was 80 °C. By decreasing the catalyst concentration, the reaction degree declined. Combined with the analysis above, it was shown that the catalytic activity of catalyst Pt-CNT was preferable compared with catalyst Pt-Al_2_O_3_ and catalyst Pt-SiO_2_ in this hydrosilylation reaction between eugenol and hydrogenterminated siloxane. This was because the CNT had a larger specific surface area and thus had a better contact with the reactants. Meanwhile, when catalyst Pt-CNT was used 4 times after recyling, it also had a high catalytic activity and the yield was still more than 90%.

### 3.2. FT–IR Analysis

**Figure 5:**
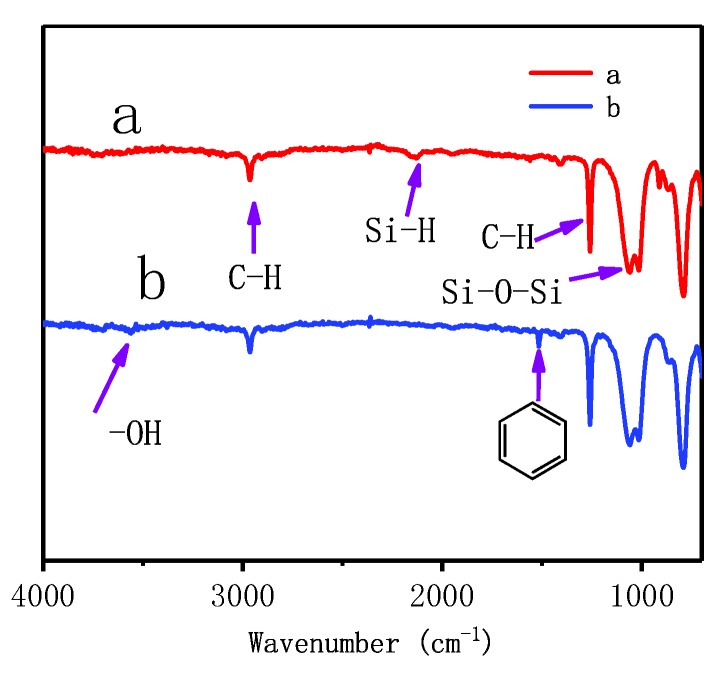
Fourier transform infrared (FT–IR) spectrum of hydrogenterminated siloxane (**a**) and biophenol terminated siloxane (**b**).

For a better understanding of the difference between hydrogenterminated siloxane and bio-phenol terminated siloxane (Sample C-2), Fourier transform infrared spectroscopy was employed to identify their functional groups. Figure 5 shows the FTIR spectrum of hydrogenterminated siloxane (a) and bio-phenol terminated siloxane (b). In both curves, an absorption peak appeared at 2900, 1300 and 1050 cm^−1^ corresponding to C–H stretching vibration, C–H in-plane bending vibration and Si–O–Si stretching vibration in turn. In curve a, a characteristic peak appeared at 2200 cm^−1^ was corresponding to Si–H stretching vibration in the molecular structure of hydrogenterminated siloxane. In curve b, the absorption peak of Si–H had disappeared, and two new characteristic peaks appeared at 3500 and 1500 cm^−1^ corresponding to O–H and benzene stretching vibration, which were derived from eugenol. It was indicated that the eugenol was connected to hydrogenterminated siloxane and bio-phenol terminated siloxane was synthesized successfully.

### 3.3. UV Analysis

**Figure 6:**
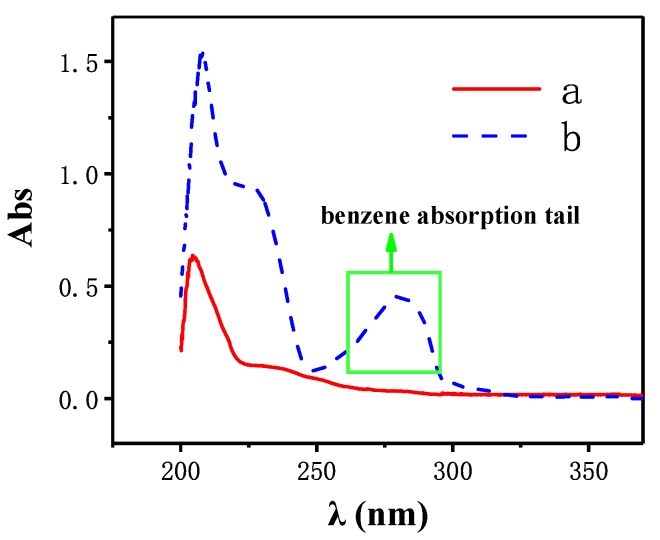
Ultraviolet–visible (UV) spectrum of hydrogenterminated siloxane (**a**) and bio-phenol terminated siloxane (**b**).

After the atoms absorb photons, the outer electrons transition from the ground state to the excited state. Different structures have different ways of transition and different wavelength range of absorbed light. A UV-visible spectrophotometer is commonly used to identify the conjugated olefin and aromatic hydrocarbon in the molecular structure according to wavelength range of absorbed light. Figure 6 shows the UV spectrum of hydrogenterminated siloxane (a) and bio-phenol terminated siloxane (b). As Figure 6 shown, in curve b, a new characteristic absorption tail appeared at the range of 240 to 280 nm were corresponding to benzene absorption tail, which were derived from eugenol. Combined with the FT–IR spectroscopy analysis above, it was demonstrated that the eugenol was connected to hydrogenterminated siloxane and bio-phenol terminated siloxane was synthesized successfully.

### 3.4. Thermal Analysis

**Figure 7:**
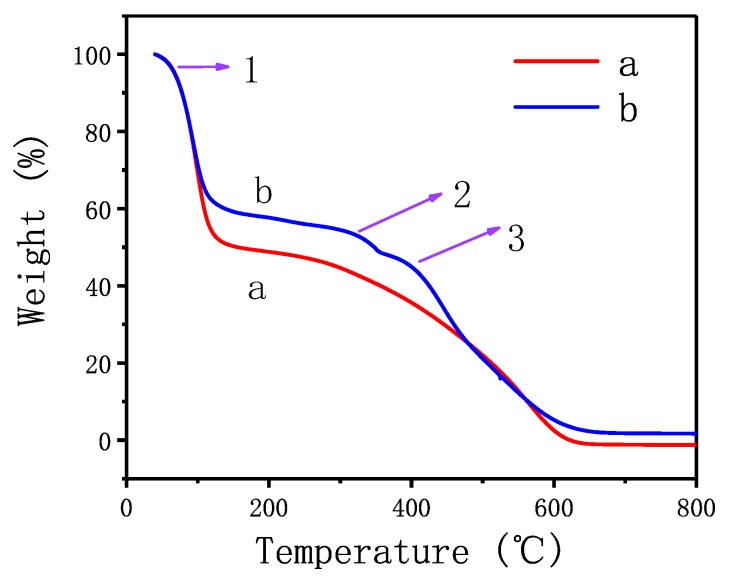
Thermogravimetric analysis (TGA) curves of hydrogenterminated siloxane (**a**) and bio-phenol terminated siloxane (**b**).

For providing evidence about effect of eugenol on enhancing the thermal stability of siloxane, the ratio of weight loss was measured. Figure 7 shows the thermogravimetric analysis (TGA) curves of hydrogenterminated siloxane (a) and bio-phenol terminated siloxane (b). In curve a, the first stage of weight loss was volatile water at the range of 80–120 °C and the second stage was mainly the decomposition of hydrogenterminated siloxane. When the ratio of weight loss reached 98%, the pyrolysis temperature was 603 °C. In curve b, the first stage of weight loss was as same as curve a and the second stage was the decomposition of siloxane and residual eugenol. The third stage of weight loss was mainly the decomposition of bio-phenol terminated siloxane. When the ratio of weight loss reached 98%, the pyrolysis temperature was 663 °C which was 60 °C higher than hydrogenterminated siloxane. It was demonstrated that the introduction of eugenol had enhanced the thermal stability of siloxane because of the benzene structure in the eugenol. 

### 3.5. Antimicrobial Activity

**Figure 8:**
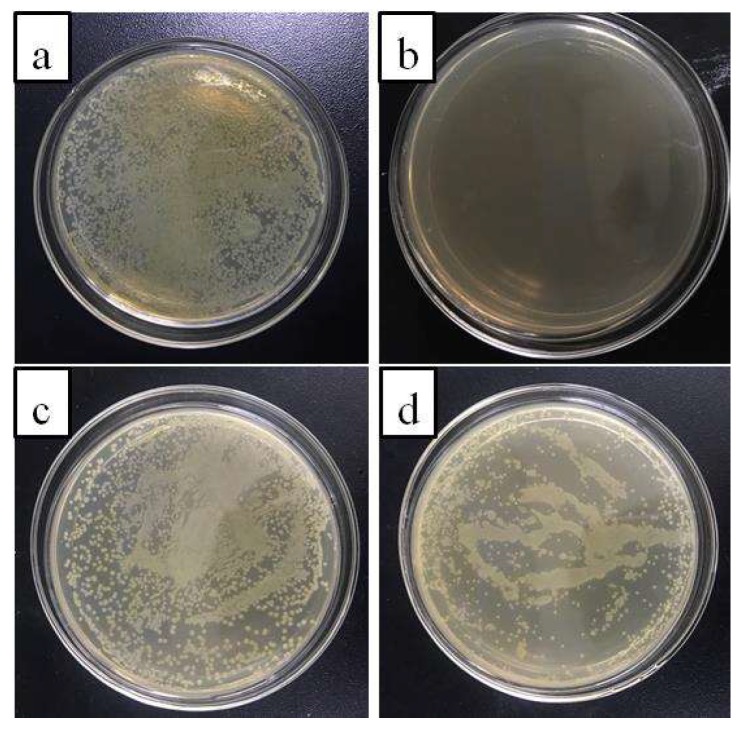
Optical images of agar plates characteristic of the antimicrobial activities of the control group (**a**), eugenol (**b**), hydrogenterminated siloxane (**c**) and bio-phenol siloxane (**d**).

Figure 8 shows optical images of the antimicrobial activities of control group (a), eugenol (b), hydrogenterminated siloxane (c) and bio-phenol terminated siloxane (d). In this assay, agar was used as nutrient solution and the *Escherichia coli* was used as model to evaluate the antimicrobial activity. In Figure 8a, it was shown that the *Escherichia coli* bacterial colonies grew quickly within the incubation time of 24 h. In Figure 8b, the presence of eugenol resulted in almost no visible *Escherichia coli* bacterial colonies and eugenol had an outstanding antimicrobial activity. Compared with Figure 8c,d, the amount of *Escherichia coli* bacterial colonies in the agar plate with the presence of bio-phenol terminated siloxane was less than the one with hydrogenterminated siloxane at the same incubation time. It was indicated that the introduction of eugenol had strengthened the antimicrobial activity of siloxane because of the characteristic structure of eugenol. The phenolic hydroxyl group was the main functional group that plays a antimicrobial activity. The ortho-methoxy group could enhance the antibacterial activity. Meanwhile, the antibacterial activity may also be related to the electrical, hydrophobic and spatial structure of bio-phenol siloxane [24]. It was shown that the bio-phenol siloxane had autonomic antimicrobial activity without the addition of antimicrobial agents.

## 4. Conclusions

Compared with catalyst Pt-Al_2_O_3_ and catalyst Pt-SiO_2_, we found the catalytic activity of catalyst Pt-CNT was preferable, because catalyst Pt-CNT has larger specific surface area and catalytic selectivity in this hydrosilylation reaction between eugenol and hydrogenterminated siloxane. When the catalyst concentration was 100 ppm, the reaction temperature was 80 °C and reaction time was 6 h, the yield reached 97% and bio-phenol terminated siloxane was synthesized successfully. Meanwhile, when catalyst Pt-CNT was used 4 times after recycling, it also had a high catalytic activity and the yield was still more than 90%. After modification with eugenol, the thermal stability of the obtained bio-phenol terminated siloxane had improved. For bio-phenol siloxane, when the ratio of weight loss reached 98%, the pyrolysis temperature was raised to 663 °C which was 60 °C higher than hydrogenterminated siloxane. Its antibacterial property against *Escherichia coli* was improved significantly. Without the addition of antimicrobial agents, the bio-phenol siloxane had autonomic antimicrobial activity because of special functional groups in the structure. Bio-phenol siloxane is expected to be applied to polymer modification, because it will enhance the thermal stability, weather resistance, hydrophobicity, and antibacterial and antioxidant qualities of the compounds.

## Figures and Tables

**Table 1:** Effect of reaction temperature on activity of catalyst.

Samples	Catalys (ppm)	Temperature (°C)	Appearance	Yield (%)
A-1	100	80	Stratified, the bottom layer was unreactive eugenol	-
A-2	100	100	Not stratified, as colorless turbid liquid	36
A-3	100	120	Not stratified, as pale yellowish turbid liquid	-
A-4	100	140	Not stratified, as yellowish turbid liquid	-
A-5	100	160	Not stratified, as yellow turbid liquid	-

**Table 2:** Effect of catalyst concentration on activity of catalyst.

Samples	Catalyst (ppm)	Temperature (°C)	Appearance	Yield (%)
A-6	50	100	Stratified, the bottom layer was unreactive eugenol	-
A-7	100	100	Not stratified, as colorless turbid liquid	-
A-8	100	100	Not stratified, as colorless turbid liquid	-
A-9	200	100	Not stratified, as colorless turbid liquid	-
A-10	500	100	Not stratified, as colorless turbid liquid	76

**Table 3:** Effect of reaction temperature on activity of catalyst.

Samples	Catalyst (ppm)	Temperature (°C)	Appearance	Yield (%)
B-1	100	60	Stratified, the bottom layer was unreactive eugenol	-
B-2	100	80	Not stratified, as colorless turbid liquid	-
B-3	100	100	Not stratified, as yellowish, little transparent liquid	66

**Table 4:** Effect of catalyst concentration on activity of catalyst.

Samples	Catalyst (ppm)	Temperature (°C)	Appearance	Yield (%)
B-4	10	80	Not stratified, as colorless turbid liquid	-
B-5	50	80	Not stratified, as colorless turbid liquid	-
B-6	100	80	Not stratified, as colorless turbid liquid	-
B-7	200	80	Not stratified, as colorless, slight transparent liquid	80

**Table 5:** Effect of reaction temperature on activity of catalyst.

Samples	Catalyst (ppm)	Temperature (°C)	Appearance	Yield (%)
C-1	100	60	Not stratified, as colorless and slight transparent liquid	-
C-2	100	80	Not stratified, as colorless transparent liquid	97
C-3	100	100	Not stratified, as yellowish and slight transparent liquid	-

**Table 6:** Effect of catalyst concentration on activity of catalyst.

Samples	Catalyst (ppm)	Temperature (°C)	Appearance	Yield (%)
C-4	30	80	Not stratified, as colorless and slight transparent liquid	-
C-5	50	80	Not stratified, as colorless and slight transparent liquid	-
C-6	100	80	Not stratified, as colorless transparent liquid	97
